# Editorial: Understanding and managing diabetic neuropathy: current perspectives and future directions

**DOI:** 10.3389/fnins.2025.1582123

**Published:** 2025-03-18

**Authors:** Paramita Basu, Pranav Prasoon, Keiichiro Susuki

**Affiliations:** ^1^Department of Anesthesiology and Perioperative Medicine, Pittsburgh Center for Pain Research, and Pittsburgh Project to end Opioid Misuse, School of Medicine, University of Pittsburgh, Pittsburgh, PA, United States; ^2^Department of Neuroscience, Cell Biology, and Physiology, Boonshoft School of Medicine, Wright State University, Dayton, OH, United States

**Keywords:** alpha-lipoic acid, brain functional connectivity, diabetic neuropathy, differential gene regulation, leptin–adiponectin, sarcopenia, Schwann cells, type 2 diabetes mellitus

Diabetic neuropathy is a heterogeneous condition that encompasses a spectrum of clinical and subclinical syndromes with varying anatomical distributions, clinical courses, and underlying pathogenetic mechanisms (Savelieff et al., 2024). The prevalence of diabetic neuropathy is high, with estimates suggesting that up to 50% of individuals with diabetes may develop the condition (Savelieff et al., 2024). The impact of diabetic neuropathy on quality of life, morbidity, and healthcare costs is significant, underscoring the importance of early detection and effective management. This Research Topic, with six original research articles and two review articles, aims to provide an overview of the current understanding of the epidemiology, pathogenesis, diagnosis, and management of this condition, with a particular emphasis on the insights gained from clinical studies.

Understanding the clinical features of diabetic neuropathy is crucial for better diagnosis, management, and treatment for this condition. In an institutional-based retrospective follow-up study design, Tilahun et al. examined diabetic neuropathy incidence and predictors among type 2 diabetes mellitus (T2DM) patients in Addis Ababa hospitals over 10 years. Of 414 T2DM participants, 23.4% developed diabetic neuropathy. Key predictors included hypertension, anemia, age, low HDL, high creatinine, and diabetic complications such as retinopathy and nephropathy. The average onset was 5 years, with a mean survival of 7 years post-diagnosis. Diabetic neuropathy is associated with a wide variety of complications. Zhang D. et al. investigated changes in brain functional connectivity (FC) in T2DM patients, comparing those with diabetic neuropathy, those without, and healthy control. Statistical analyses identified group differences and linked FC changes to clinical measures, showing that diabetic neuropathy is associated with reduced FC in brain regions essential for motion and motor control (Zhang D. et al.). These findings suggest that diabetic neuropathy may impair movement-related cognitive function, highlighting the need for comprehensive assessments and personalized treatments to enhance patient quality of life. Fang et al. explored the relationship between sarcopenia and diabetic neuropathy in older patients with T2DM. Mendelian randomization analysis suggested a partial causality between diabetic neuropathy and the clinical traits of sarcopenia. In addition, the cross-sectional study suggested that appendicular skeletal muscle mass index and the 5-time chair stand test were linked to diabetic neuropathy. These data highlight the importance of screening for sarcopenia in patients with diabetic neuropathy.

Understanding the underlying genetic, immunological, or metabolic factors associated with diabetic neuropathy is crucial for the development of effective diagnostic and therapeutic strategies. Zhang Y. et al. investigated diabetic neuropathy using bioinformatic analysis to identify differentially expressed genes (DEGs) in human and mouse samples. Researchers found several hub genes associated with immune dysregulation in diabetic neuropathy, revealing shared DEGs between species. The study emphasized the significance of immune cell infiltration and suggests these findings could provide new diagnostic and therapeutic targets for diabetic neuropathy. Chen et al. found a relationship between circulating adiponectin and leptin levels and the risk of diabetic neuropathy in T2DM patients. Logistic regression models were used to analyze data from 198 diabetic neuropathy patients and 205 diabetic controls, and they found that lower adiponectin and higher leptin levels were significantly associated with an increased risk of diabetic neuropathy (Chen et al.). These findings suggest that adipokines could serve as potential biomarkers for identifying at-risk individuals, emphasizing the need for further research into the underlying mechanisms. In a review article, Sango et al. explored the use of immortalized Schwann cell lines to study diabetic neuropathy pathogenesis and therapy. This highlights how hyperglycemia-related abnormalities in Schwann cells contribute to the development and progression of diabetic neuropathy, focusing on metabolic changes and stress responses. This Research Topic also discusses various pathogenic factors and therapeutic strategies aimed at alleviating the effects of diabetic neuropathy.

Early diagnosis and effective management strategies are crucial for mitigating the adverse effects of this condition on a patient's wellbeing and quality of life. Liu et al. developed a nomogram model to predict the risk of diabetic neuropathy in patients with diabetes. By analyzing data from 1,185 individuals, the researchers identified seven significant risk factors, including age, hip circumference, fasting plasma glucose, fasting C-peptide, postprandial C-peptide, albumin, and blood urea nitrogen. The model demonstrated good predictive ability, providing a practical tool for clinicians to identify high-risk individuals early. This could enhance treatment outcomes in various clinical settings, particularly in cases where medical resources are limited. Finally, Atmaca et al. highlighted the importance of early detection, accurate diagnosis, and personalized treatment. It emphasized the need for greater physician awareness, particularly among first-contact clinicians, to ensure timely intervention. A structured screening and diagnostic algorithm recommend evaluating diabetic neuropathy in at-risk patients with prediabetes or diabetes by incorporating laboratory testing and neurological referrals for atypical cases. The study advocated individualized treatment approaches targeting specific mechanisms rather than a universal strategy. Alpha-lipoic acid was identified as a promising first-line antioxidant therapy for diabetic neuropathy, and its dual antioxidant effects might help alleviate diabetic neuropathy-related conditions. However, research gaps persist in understanding disease progression and treatment efficacy (Atmaca et al.). This study calls for robust multicenter trials and biomarker discovery to enable more personalized therapies and emphasizes the urgent need to improve awareness, diagnostics, and targeted treatments to manage diabetic neuropathy effectively.

We hope the reader will find this Research Topic a valuable reference for diabetic neuropathy, which has long been a subject of intense research and clinical focus. In conclusion, these articles offer new insights into the underlying mechanisms and development of innovative therapeutic approaches for diabetic neuropathy.
